# How Chinese College Students Coped with COVID-19 Burnout: A Cross-Sectional Study of the Mediating Effect of Family Support and Interaction Quality

**DOI:** 10.3390/bs15060736

**Published:** 2025-05-26

**Authors:** Jia Zhuang, Susan Xiqing Su

**Affiliations:** 1 Department of Applied Social Sciences, The Hong Kong Polytechnic University, Hong Kong SAR, China; 2 College of International Education, Hong Kong Baptist University, Hong Kong SAR, China

**Keywords:** psychological wellbeing, COVID-19 burnout, family interaction quality, family support, Chinese university students

## Abstract

While it is widely acknowledged that the COVID-19 pandemic posed unprecedented challenges to the mental wellbeing of college students globally, particularly among those studying abroad, little is known about how students navigated the pandemic-related challenges and maintained their psychological wellbeing. This study identifies Chinese college students’ coping mechanisms for COVID-19 burnout by investigating the mediating effects of high-quality family interaction and family support on the relationship between COVID-19 burnout and psychological wellbeing. A cross-sectional survey was conducted across four regions—Shanghai, Hong Kong, Macau, and London—encompassing a diverse sample of Chinese university students (*N* = 971), representing a wide range of demographics. Structural equation modelling was applied to test the mediation model. The results revealed a significantly positive correlation between university students’ psychological wellbeing and the family interaction quality and support received. In addition, family interaction quality and support were significantly negatively correlated with the students’ COVID-19 burnout. The mediation analysis further suggested that family interaction quality and support mediated the association between the students’ COVID-19 burnout and psychological wellbeing. These findings have contributed to the literature by addressing the underexplored external factors affecting psychological wellbeing, offering vital insights for informing targeted interventions aimed at supporting students during public health crises.

## 1. Introduction

In recent decades, the global community has faced unprecedented challenges and consequences arising from pandemic outbreaks. These events, characterized by the rapid spread of infectious diseases on a global scale, not only strain regional and international public health systems but also threaten individuals’ mental wellbeing (Voltmer et al., 2021). For example, in the early stage of the COVID-19 pandemic, a survey of participants from 194 cities in China found that nearly one-third of the participants reported moderate to severe anxiety symptoms (C. Wang et al., 2020). Another study with French college students found that the prevalence of depression, anxiety, and stress reached 40% during the COVID-19 pandemic (Essadek & Rabeyron, 2020).

College students represent one of the most vulnerable populations during such public health crises. Their mental health was adversely affected by a myriad of stressors and uncertainties stemming from the COVID-19 pandemic (Campbell et al., 2022). The implementation of social distancing measures and the closure of educational institutions forced students to transition abruptly from traditional face-to-face learning to remote or hybrid modalities. This shift necessitated not only adequate access to digital devices and reliable internet connectivity for online learning but also required students to quickly adapt to new technologies and learning environments. Challenges included navigating online platforms for educational purposes and experiencing diminished interaction with peers and instructors (Shek, 2021). These factors collectively put students at remarkable risk of COVID-19 burnout and posed a considerable threat to their psychological wellbeing (Villani et al., 2021).

Burnout is defined as a state of physical, emotional, and mental exhaustion caused by prolonged and excessive stress (Sveinsdóttir et al., 2021; Toubasi et al., 2023). It is a situation that is different from specific clinical psychological disorders, such as anxiety and depression (Sun et al., 2022). Burnout consists of three dimensions: While emotional exhaustion is the feeling of being drained, fatigued, and overwhelmed by demands, depersonalization refers to a cynical or detached attitude toward responsibilities and obligations. The last, reduced personal accomplishment, implies a feeling of ineffectiveness and a decline in self-esteem, leading individuals to feel unproductive or unfulfilled. COVID-19 burnout refers to the feelings of exhaustion, stress, and disillusionment experienced by individuals due to the challenges brought about by the pandemic and its related measures (Lau et al., 2022; Yıldırım & Solmaz, 2022). As for students, COVID-19 burnout was not caused by the pandemic alone but also encompassed a wide range of academic and social factors, such as academic pressure, social isolation, and uncertainties about the future (Abraham et al., 2024). A study in Spain found that the prevalence of burnout among university students reached 21% during the COVID-19 pandemic (Aguayo-Estremera et al., 2023). In China, nearly 40% of nursing students were found to have some degree of burnout during COVID-19 (J. Wang et al., 2021).

Empirical studies have consistently suggested a positive correlation between COVID-19 burnout and psychological distress among college students across the globe (Chen et al., 2024; Martínez-Líbano et al., 2021; Merino-Godoy et al., 2022; Sun et al., 2022). In China, the study by Chen et al. (2024) examined the interrelationships among COVID-19 prevention burnout, depression, anxiety, and psychological flexibility based on a sample of 1837 Chinese university students. The results suggested that students with higher levels of COVID-19 burnout tended to experience higher levels of depression and anxiety. This result was echoed by another study, which was conducted by Sun et al. (2022) and was based on a sample of 388 college students from Nanjing City. The results revealed that COVID-19 burnout contributed to students’ psychological distress, such as depression, anxiety, and somatization. These two studies further explored the mechanism behind the relationship between COVID-19 burnout and psychological distress by incorporating two internal factors into the models and determining the mediating effects of resilience and psychological flexibility on the association between COVID-19 burnout and psychological distress. Furthermore, among the college students studied, those studying abroad were reported to have been more vulnerable to COVID-19-related burnout since they were inclined to encounter challenges in cultural adaptation, the destruction of social bonding in both home and host cities, financial strain, and immigration regulations (Y. Wu et al., 2024).

Yet, while scholarly efforts have been made in exploring students’ internal factors when explaining the correlation between their COVID-19 burnout and psychological distress, little is known about the possible mediating effect of external support. Such an investigation might be theoretically supported by the ecological system theory and a multisystemic resilience perspective. First, an ecological system perspective (Bronfenbrenner, 2000) stresses that individuals’ development is affected by multifarious factors from their micro-, meso-, and macro-environment. As for university students, their way of coping with COVID-19 burnout was not only shaped by their inner strength in terms of internal resilience and psychological flexibility (Chen et al., 2024), but also by the support and resources received from their family, school, and peer system (Shek et al., 2023; Haleemunnissa et al., 2021; Karikari et al., 2021). For instance, in a study with Hong Kong university students, researchers observed that, while pandemic-related stress positively predicted psychological morbidity, environmental factors, including healthy family functioning, peer support, and a supportive community atmosphere, would mitigate such negative effects and enhance students’ wellbeing (Shek et al., 2023). Along the same lines, a multisystemic resilience perspective has underscored the significance of multiple external systems in fostering the resilience of individuals and the protective effects provided (Ungar, 2021; Rutter, 2023). It suggested that, in addition to individuals’ internal resilience assets, resources and support from external systems, coupled with the dynamic interplays among individuals’ self-system and multiple external systems, contribute to individuals’ overall resilience, empowering them to triumph over adversities.

In the context of COVID-19, Prime et al. (2020) developed a conceptual framework to illustrate the significance of the family system in mitigating the negative influences of COVID-19 and protecting adolescents’ physical health, their psychological and social wellbeing, and their educational development. First, the COVID-19 pandemic has profoundly affected family dynamics, creating remarkable uncertainties that disrupt social interactions and support systems. These disruptions not only threaten the wellbeing of children but also impact caregivers, leading to increased psychological distress, parenting challenges, and mental health symptoms. Based on this, we hypothesized the following:

**H1a.**  *COVID-19 burnout would be negatively correlated with university students’ family interaction quality.*

**H2a.**  *COVID-19 burnout would be negatively associated with university students’ family support.*

Nonetheless, as underlined in the Family Systems Theory, a family operates as an interconnected system, where all family members are interrelated (Walsh, 2015; Weiss et al., 2013). The behavior of an individual family member influences the entire family system, creating ripple effects throughout. The overall family atmosphere might affect the psychological status of individual members. When confronted with external adversities, the family system might empower the individual members to cope with the difficulties, safeguarding their wellbeing and development (Prime et al., 2020; Masten & Narayan, 2012). Quality communications and connections between parents and their children, coupled with the support and resources from the family system or the family’s access to external resources and support, are able to mitigate these negative effects and help children in a smooth adjustment. While effective communication defines the function of the family as a system, as well as the wellbeing of individual members (Buehler et al., 2024), family support provides emotional buffering, practical resources, and guidance regarding coping strategies (Pinkerton & Dolan, 2007). Based on this, we hypothesized the following:

**H1b.**  *Family interaction quality would be positively correlated with students’ psychological wellbeing.*

**H2b.**  *Family support would be positively associated with students’ psychological wellbeing.*

**Figure 1:**
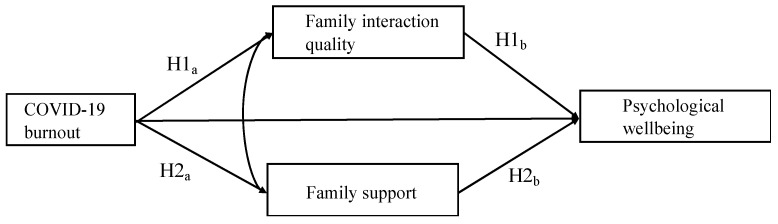
The conceptual model about the mediation relationships among COVID-19 burnout, family interaction quality, family support, and psychological wellbeing.

Yet, while the theoretical framework underlines the significant role played by family interaction and family support in students’ coping strategies in relation to COVID-19 burnout and their psychological wellbeing, no study has statistically tested their mediating effect on the correlation between COVID-19 burnout and psychological wellbeing. The current study is one of the early endeavors to explore the mechanism behind the correlation between COVID-19 burnout and psychological wellbeing among college students, with a consideration of their external factors. It contributes to the literature by examining the role of family interaction quality and family support in mediating the association between COVID-19 burnout and psychological wellbeing among Chinese university students across four regions. This study is significant as it highlights the critical role of family dynamics in mitigating the adverse effects of COVID-19 burnout on university students’ psychological wellbeing. By identifying the mediating effects of family interaction quality and support, it is believed that the findings provide valuable insights for developing targeted interventions, which could enhance students’ resilience and mental health during future public health crises. Figure 1 presents the conceptual model and four hypotheses of this study.

## 2. Methodology

### 2.1. Data and Participants

To test the mediating effect of family interaction quality and family support on the association between COVID-19 burnout and psychological wellbeing, we conducted a cross-sectional study on mainland Chinese university students who left their hometown to study in Shanghai, Hong Kong, Macau, and London. We obtained ethical approval from the Institutional Review Board of the corresponding author’s institution. Applying a convenient sampling method, the research team approached scholars from the universities in the aforementioned four regions and invited them to disseminate the QR code and the weblink to the online questionnaire to students, not only from their affiliated universities, but from all universities in their region. With informed consent displayed at the beginning of the survey package, we invited student participants from the four regions to complete an online survey in either Qualtrics or WeChat, the most common social media utilized among Chinese people. Each survey took 15 to 20 mins. Students’ participation was completely voluntary. They could withdraw anytime without any consequences.

The survey was conducted between March and October 2023. A total of 971 university students were recruited from four cities. It is worth noting that, while the pandemic had passed its peak, its profound influence on individuals’ lives and work persisted at the time our data were collected. The psychological effects, such as anxiety and stress, persisted, alongside lasting changes in school dynamics due to the shift to blended learning modalities. Likewise, concerns about future job prospects and health safety influenced their educational choices and mental wellbeing (Dave et al., 2024). Table 1 presents the detailed demographic characteristics of the sample. There were more female participants (57.7%) than male participants (42%) in this study. Over half of the total participants were pursuing their bachelor’s degree when surveyed, with another 27.4% enrolled in taught master’s programs. As for their destination of study, the proportion among the four regions was almost even, with slightly more students from London (28.1%). Lastly, nearly half of the parents of the participants in this study had completed their tertiary education.

### 2.2. Measures

COVID-19 burnout. The Chinese version of the Burnout Frequency Scale-5 (BFS-5) was developed by Lau et al. (2022) to measure the frequency of COVID-19 burnout and emotional fatigue. A confirmatory factor analysis, based on a sample of 1087 individuals from Hong Kong, verified its validity and reliability (α = 0.90). Participants were asked to rate their feelings as follows: (1) emotionally exhausted because of the COVID-19 pandemic and the preventive measures, (2) stressed by adhering to the COVID-19 preventive measures, (3) irritable and have a shortening fuse with the COVID-19 preventive measures, (4) hopeless as the COVID-19 pandemic continues despite preventive measures, and (5) being trapped in the city due to the travel bans and restrictions during the COVID-19 pandemic. Each item was rated on a 7-point Likert scale, ranging from “strongly disagree (coded as 1)” to “strongly agree (coded as 7)”. The total score of the scale ranged from 5 to 35, with a higher score indicating a greater frequency of COVID-19 burnout. The alpha coefficient of the scale was 0.818 in this study, indicating good reliability.

Psychological wellbeing. Students’ psychological wellbeing was measured using the Flourishing Scale (FS-8), which is a unidimensional instrument that is widely used to assess self-perceived emotional wellbeing in key areas, such as relationships, self-esteem, purpose, and optimism (Schotanus-Dijkstra et al., 2016; Diener & Chan, 2011). The scale employs a 7-point Likert scale to gauge participants’ levels of agreement with eight statements, ranging from “strongly disagree (coded as 1)” to “strongly agree (coded as 7)”. Sample items are as follows: “I lead a purposeful and meaningful life”, “My social relationships are supportive and rewarding”, and “I am optimistic about my future”. The FS demonstrates strong psychometric properties and has been validated within Chinese populations, with Cronbach’s alpha coefficient ranging from 0.88 to 0.93 (Tong & Wang, 2017; Zhou & Huo, 2022). The scale was applied among Chinese international university students (Dong & Li, 2024). Its reliability was found to be high (α = 0.88). In this study, the reliability of the scale was excellent (α = 0.902). The total score of the scale ranges from 8 to 56, with higher scores on the FS reflecting greater psychological resources, strengths, and a higher degree of subjective wellbeing and happiness (Diener & Chan, 2011).

Family interaction quality. The students’ family interaction quality was assessed using an independent subscale of the Family Quality of Life scale, which was developed by Poston et al. (2003). The subscale consists of six items. Example items include the following: “My family members frequently feel loved and accepted by each other” and “My family members enjoy gathering time”. The Chinese version of the family interaction quality subscale has demonstrated strong validity across various Chinese family contexts (Liu et al., 2025; Chiu et al., 2017), with Cronbach’s alpha coefficients ranging from 0.88 to 0.93. The alpha coefficient of the scale in this study was 0.901, indicating excellent reliability. This scale employs a 7-point Likert format, ranging from “strongly disagree (coded as 1)” to “strongly agree (coded as 7)”. The total score of the subscale ranges from 6 to 42, with a higher overall score reflecting a higher quality of family relationships, a more positive family interactional environment, and improved communication among family members.

Family support. The Family Emotional Wellbeing scale is an independent subscale of the Family Quality of Life framework, which was developed by Poston et al. (2003). It evaluates the connectedness of family members, as well as the mutual support within the family system. The Chinese version of the subscale has been validated across various Chinese family contexts (e.g., Liu et al., 2025; Chiu et al., 2017), with a Cronbach’s alpha ranging from 0.69 to 0.73. The reliability of the scale in this study was acceptable (α = 0.778). The scale consists of four items. Example items from the subscale include the following: “My family has the support we need to relieve stress”, “My family has outside help available to us to take care of special needs of all family members”. The scale employs a 7-point Likert scale, with anchors ranging from “strongly disagree (coded as 1)” to “strongly agree (coded as 7)”. The total score of the subscale ranges from 4 to 28, with a higher score indicating a greater level of family support.

Covariates. The parental educational level might have affected the family communication quality and was, thus, controlled in the model (H. Park, 2008). Other covariates included the students’ gender, educational level, parents’ educational level, and their place of study.

### 2.3. Analytic Approach

To test the mediating effect of family interaction quality and family support on the association between COVID-19 burnout and psychological wellbeing among Chinese university students, structural equation modelling (SEM) was instructed using Stata 17, followed by an estimation of the correlation among the variables. In the constructed mediation model, the students’ COVID-19 burnout was entered as the predicting variable, while family interaction quality and family support were the two mediators, and psychological wellbeing was the outcome variable. In addition, students’ gender, age, education, place of study, and parental educational level were controlled in the model. The measurement and structural models were considered to fit the data if the chi-square *p*-value was below 0.05, the root mean square error of approximation (RMSEA) estimate was less than 0.08, the standardized root mean square residual (SRMR) was below 0.05, and the comparative fit index (CFI) was above 0.9 (Hu & Bentler, 1995). Furthermore, the statistical significance of the indirect effects was evaluated using the Monte Carlo method, based on 5000 bootstrap samples (Preacher & Selig, 2012).

## 3. Results

### 3.1. Descriptive Statistics

Table 2 presents the descriptive statistics and bivariate correlations among COVID-19 burnout, family interaction quality, family support, and psychological wellbeing. First, the students’ COVID-19 burnout showed a weak negative correlation with their family interaction quality (*r* = −0.16, *p* < 0.001), family support (*r* = −0.10, *p* = 0.0016), and psychological wellbeing (*r* = −0.13, *p* < 0.001). Second, family interaction quality (*r* = 0.55, *p* < 0.001) and family support (*r* = 0.55, *p* < 0.001) were moderately positively correlated with psychological wellbeing. Lastly, the two familial factors demonstrated a strong positive correlation with each other (*r* = 0.70, *p* < 0.001). This suggested that higher quality interactions within the family are likely to enhance the perception of support among students. When family members engage in meaningful communication and activities, it fosters a sense of connection and understanding, which can strengthen the overall support system (Walsh, 2015). Likewise, when students perceive strong family support it may encourage more positive and frequent interactions, creating a reinforcing cycle.

### 3.2. Mediation Results

**Figure 2:**
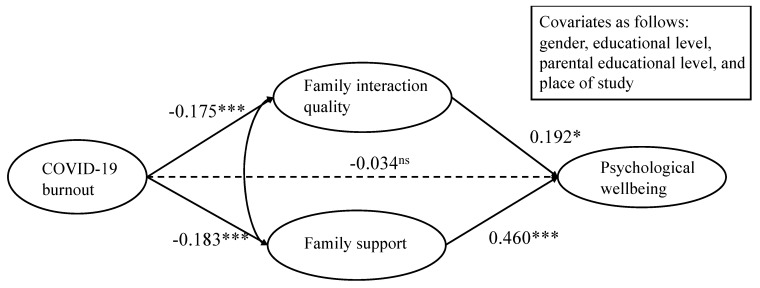
Results of mediation analysis. **Notes.** * *p* < 0.05; *** *p* < 0.001, ns non-significant.

To test our hypotheses, structural equation modelling was used to determine whether the association between COVID-19 burnout and psychological wellbeing would be mediated by family interaction quality and family support. First of all, the model showed a good fit to the data, with χ^2^ (327) = 1274.71; *p* < 0.001; CFI = 0.917; SRMR = 0.060; RMSEA = 0.057; and 90% CI for RMSEA [0.054, 0.060]. As shown in Figure 2 and Table 3, the students’ COVID-19 burnout was negatively correlated with their family interaction quality (*β* = −0.175, *p* < 0.001) and family support (*β* = −0.183, *p* < 0.001), supporting H_1a_ and H_2a_. The students’ family interaction quality was positively correlated with their psychological wellbeing (*β* = 0.192, *p* < 0.033), supporting H_1b_. Notably, family support demonstrated a robust and significant correlation with psychological wellbeing (β = 0.460, *p* < 0.001), supporting H_2b_.

In addition, to quantify and conduct an inferential test for the mediating effect, the Monte Carlo method was applied, based on 5000 bootstrap samples. The results indicated that the mediating effects of family interaction quality (*b* = −0.044; *p* = 0.01; and CI [−0.080, −0.014]) and family support (*b* = −0.076; *p* = 0.001; and CI [−0.125, −0.035]) were statistically significant. Altogether, the results suggested that family interaction quality and family support mediated the relationship between COVID-19 burnout and psychological wellbeing. Furthermore, the direct effect of COVID-19 burnout on psychological wellbeing was nonsignificant after considering the mediating effects of family interaction quality and family support, which suggested the correlation between COVID-19 burnout and psychological wellbeing was fully mediated by family interaction quality and family support.

## 4. Discussion

Empirical evidence has suggested that public health crises cause burnout in university students, which further poses a considerable threat to their mental health. While existing scholarly efforts have extended to explaining the mechanisms behind the correlation between public-health-related burnout and students’ psychological wellbeing, internal resilience, and psychological flexibility (Chen et al., 2024), the possible mediating effect of factors from students’ external systems remains understudied. This study is one of the early endeavors being made to fill this knowledge gap. Drawing upon a cross-sectional approach, we explored the interrelationship among Chinese university students’ COVID-19 burnout, family interaction quality, family support, and psychological wellbeing. The results uncovered the mediating effect of family interaction quality and family support on the correlation between COVID-19 burnout and psychological wellbeing among Chinese university students.

First, we found that students with higher levels of COVID-19 burnout reported receiving less family support and experiencing lower quality family interactions. This relationship may stem from two main factors, as follows: First, COVID-19 burnout might have reduced the frequency of family interactions and hindered mutual support. Students often faced heightened concerns about the virus, abrupt disruptions in their social networks, and social isolation due to preventive measures. These factors significantly limited their ability to communicate physically and emotionally with family members, thereby restricting the support they received (Andrade et al., 2023; Salmela-Aro et al., 2022). Second, COVID-19 burnout may have negatively impacted the quality of their family interactions. The stress and anxiety associated with the pandemic could have led students to engage in superficial or contentious discussions. Both students and their family members may have struggled with their emotional challenges, resulting in misunderstandings and conflicts rather than supportive exchanges (Feinberg et al., 2021; M. Wu et al., 2020). This decline in interaction quality could have left students feeling unsupported and disconnected, exacerbating their sense of isolation (Hall & Zygmunt, 2021).

Although COVID-19 burnout might have led to decreased family support and reduced family interaction quality, our study further revealed that family support and family interaction quality positively contributes to students’ psychological wellbeing. Family system theory suggests that effective communication patterns are crucial to the function of the family as a system, as well as to the wellbeing of each individual member (Buehler et al., 2024). While open and honest communication fosters mutual support, poor communication might lead to misunderstandings and conflict (Elgar et al., 2013). Studies have shown that family communication quality significantly impacts psychological wellbeing, particularly during stressful times, such as the COVID-19 pandemic (Prime et al., 2020). Individuals with quality family interactions tend to engage in adaptive coping strategies, which might alleviate the psychological distress associated with burnout (Geçer & Yıldırım, 2023; Gutierrez Ticona et al., 2024). For example, a longitudinal study with 502 Mexican students found that a positive family environment not only decreased students’ general stress but also enhanced their subjective wellbeing (Gaxiola Romero et al., 2022).

Family support was of particular importance to university students’ psychological wellbeing during COVID-19. The reasons behind this might be threefold: First, family members provide emotional buffering, helping students process their feelings of burnout and reducing anxiety, which enhances mental health (Koutsimani & Montgomery, 2023). A strong family support system also fosters social connections, reducing feelings of isolation and promoting a sense of belonging. Second, families offer practical resources, such as financial assistance or help with academic responsibilities, alleviating stressors that contribute to burnout (Mariani et al., 2020). Scholars further stressed that family support was not only confined to the support from family members but extended to support from external bodies and the family as a whole (Poston et al., 2003). Third, supportive families might guide students in developing effective coping strategies for managing stress, encouraging healthier responses to burnout (Liu & Cao, 2022). A nurturing family environment motivates students to engage in self-care and seek professional help when necessary, further improving their psychological wellbeing. For instance, a study in Henan, China, found that family support predicted university students’ coping strategies regarding COVID-19, which was further positively linked to their mental health (Yang et al., 2022). Another study with university students from Malaysia drew a similar conclusion. The students who received more support from their families and peers demonstrated a higher quality of life during the COVID-19 pandemic (Abdullah et al., 2021). These findings are relevant as they suggest that fostering quality interactions and support within families could be a vital strategy for improving psychological wellbeing among university students, particularly during challenging times. By focusing on enhancing family communication and support systems, interventions could effectively mitigate the negative effects of stressors such as COVID-19 burnout, promoting psychological resilience and better mental health outcomes.

The findings of this study carry substantial implications for enhancing the psychological wellbeing of Chinese university students, particularly in the context of public crises. As the research has revealed, the quality of family interaction and the level of family support play crucial mediating roles in the relationship between COVID-19 burnout and psychological wellbeing. This insight suggests that fostering healthy family dynamics and effective communication might serve as vital protective factors, which mitigate the adverse effects of burnout. It is, thus, important for educational institutions, policymakers, and mental health practitioners to leverage these insights to develop comprehensive support programs that emphasize the importance of family engagement. Initiatives could include providing workshops aimed at improving family communication skills, creating platforms for students and their families to connect more effectively, and providing resources that promote understanding and empathy within family units. Given that parents and university students are always physically separated, developing online or web-based parenting programs may provide an effective solution. A universal web-based parenting program has significantly enhanced parental support and skills among participating parents in Latin American countries (Suárez et al., 2018), demonstrating the potential of virtual platforms to equip distant, working parents with improved parenting practices.

Furthermore, raising awareness about the psychological impacts of COVID-19 burnout among students and their families might empower individuals to recognize the signs of distress and seek support when needed. Universities should consider offering workshops focused on family communication skills, such as teaching conflict resolution and supportive dialogue techniques to strengthen familial interactions. Likewise, by integrating family support systems into mental health strategies, universities would be able to cultivate a more resilient student population, one that is better equipped to cope with the stresses of academic life and external crises. This holistic approach would not only address immediate mental health needs but would also strengthen familial bonds, contributing to a supportive environment that enhances overall wellbeing. In addition, establishing peer support networks could also be beneficial, allowing students to share experiences and coping strategies within a community setting. Furthermore, awareness campaigns promoting mental health education would empower students and their families to recognize the signs of distress and to seek appropriate support. Ultimately, the study advocates for a systemic response that recognizes the interconnectedness of family dynamics and individual mental health, setting the stage for more effective interventions in the future.

While this study has provided valuable insights into the mediating effects of family interaction quality and family support on the relationship between COVID-19 burnout and psychological wellbeing among Chinese university students, several limitations must be acknowledged. First, the cross-sectional design of the study restricted its ability to draw causal inferences regarding the relationships among variables. Although associations were identified, it remains unclear whether family support and interaction quality directly influence psychological wellbeing or if other underlying factors contribute to these dynamics. This ambiguity limited our ability to make informed recommendations for interventions aimed at enhancing student wellbeing. Second, the reliance on self-reported measures may have introduced response bias, as students might have overestimated or underestimated their levels of burnout, family support, and psychological wellbeing. This subjectivity could have affected the reliability of the data collected. Likewise, the measure of COVID-19 burnout has not been validated among Chinese international students. This limitation raises concerns about the reliability and applicability of the findings, as the experiences and cultural contexts of students may vary significantly according to their place of study.

Additionally, the study’s focus on a specific demographic—Chinese university students—has limited the generalizability of the findings to other populations or cultural contexts. Variations in family structures, social support systems, and coping mechanisms across different cultures may influence the dynamics explored in this research. As such, the insights gained may not be applicable to students in different cultural or geographical contexts, potentially overlooking important factors that contribute to psychological wellbeing. Moreover, the study did not account for other potential external factors, such as support from peers, school, and community systems. These external factors could play a crucial role in buffering against the negative effects of COVID-19 burnout and neglecting them may have resulted in an oversimplified understanding of the interplay between support systems and wellbeing during public health crises. Finally, it is worth noting that the study was conducted after the peak of COVID-19. The students’ perceptions and experiences of COVID-19 burnout, as well as its effect on their psychological wellbeing, might have been different at the time of the survey than during the peak days of the pandemic. Therefore, the findings may not have fully captured the intensity of the burnout experienced during the peak period, nor the immediate psychological impacts associated with it.

## 5. Conclusions

This study has highlighted the critical role of family interaction quality and support in mediating the relationship between COVID-19 burnout and the psychological wellbeing of Chinese university students studying in four different cities: Shanghai, Macau, Hong Kong, and London. Our findings indicated that higher levels of burnout are associated with decreased family support and interaction quality, which, in turn, negatively impact students’ mental health. Conversely, fostering strong family communication and support systems might significantly enhance students’ psychological wellbeing, particularly during public health crises. These insights underscore the importance of addressing familial dynamics in mental health interventions, suggesting that targeted strategies to improve family relationships could mitigate the adverse effects of burnout and promote better mental health outcomes for university students.

## Figures and Tables

**Table 1:** Demographic characteristics of the sample.

Demographic Features		Frequency (*N* = 971)	Percentage (%)
Gender			
	Male	408	42.0
	Female	560	57.7
	Others	3	0.3
Educational level			
	Bachelor’s Year 1	110	11.3
	Bachelor’s Year 2	187	19.3
	Bachelor’s Year 3	149	15.3
	Bachelor’s Year 4	97	10.0
	Bachelor’s Year 5	21	2.2
	Taught master’s	266	27.4
	Research postgraduate	141	14.5
Place of study			
	London	273	28.1
	Hong Kong	208	21.4
	Macau	246	25.4
	Shanghai	243	25.1
Father’s education level			
	Primary school	63	6.5
	Junior high school	175	18.0
	Senior high school	251	25.9
	Tertiary	481	49.6
Mother’s educational level			
	Primary school	88	9.1
	Junior high school	172	17.7
	Senior high school	271	27.9
	Tertiary	439	45.3

**Table 2:** Means, standard deviation, and bivariate correlations among variables.

	Min.	Max.	Mean	SD	1	2	3	4
1. COVID-19 burnout	5	35	18.79	7.64	-			
2. Family interaction quality	6	42	33.37	7.15	−0.16 ***	-		
3. Family support	4	28	20.80	4.79	−0.10 **	0.70 ***	-	
4. Psychological wellbeing	8	56	44.24	8.13	−0.13 ***	0.55 ***	0.55 ***	-

Notes: ** *p*-value < 0.01; *** *p*-value < 0.001.

**Table 3:** Results of path analysis in the mediation model.

	* β *	SE	*p*-Value
Outcome: psychological wellbeing			
COVID-19 burnout	−0.034	0.031	0.277
Family interaction quality	0.192	0.090	0.033
Family support	0.460	0.092	<0.001
Education	0.053	0.030	0.077
Gender	−0.051	0.030	0.095
Parental education	0.020	0.030	0.500
Place of study	−0.15	0.030	0.623
Outcome: family interaction quality			
COVID-19 burnout	−0.175	0.036	<0.001
Education	0.049	0.035	0.165
Gender	−0.091	0.035	0.008
Parental educational level	0.161	0.034	<0.001
Place of study	0.070	0.035	0.045
Outcome: family support			
COVID-19 burnout	−0.183	0.038	<0.001
Education	0.001	0.037	0.972
Gender	−0.171	0.036	<0.001
Parental education level	0.163	0.036	<0.001
Place of study	0.055	0.037	0.132

## Data Availability

The data that support the findings of this study are available upon request to the corresponding author of the paper.
